# Increasing uptake of physical health checks for people living with severe mental illness: a systematic review

**DOI:** 10.3399/BJGP.2024.0826

**Published:** 2025-11-18

**Authors:** Elizabeth Emsley, Sarah A Sullivan, Elizabeth Rose-Innes, Emma Sidebotham, Clare E French

**Affiliations:** 1 South West Public Health Training Programme, Severn Postgraduate Medical Education School of Public Health, Bristol, UK; 2 Population Health Sciences, Bristol Medical School, University of Bristol, Bristol, UK; 3 NIHR Health Protection Research Unit in Behavioural Science and Evaluation, University of Bristol, Bristol, UK

**Keywords:** adult psychiatry, general practice, physical health promotion, primary health care, public health, severe mental illness

## Abstract

**Background:**

People living with severe mental illness (SMI) can experience greater risk of premature mortality than the general population with preventable physical ill health a major contributor. Physical health checks are a strategy to improve physical health but uptake is variable.

**Aim:**

To collate and assess the published evidence on the effectiveness of interventions to increase access to, and uptake of, physical health reviews for people living with SMI.

**Design and setting:**

This was a systematic review.

**Method:**

Three databases (Medline, CINAHL, and PsycINFO) were searched for studies evaluating interventions aiming to increase access to and uptake of physical health checks for people with SMI. Searches were conducted during October and November 2024 for studies published from 2000 onwards. Studies were included that reported on outcomes of uptake or receipt of physical health checks or screening, including cancer screening. Both randomised controlled trials (RCTs) and comparative non-randomised studies were eligible. Primary, secondary, and tertiary care settings were included. Studies from high-income countries were also included. Risk of bias was assessed using version 2 of the Cochrane risk-of-bias tool for randomised trials (RoB2), an adaptation of the Risk of Bias in Non-randomised Studies of Exposures (ROBINS-E), and Risk of Bias in Non-randomised Studies — of Interventions (ROBINS-I) tools. Data synthesis involved an effect direction plot.

**Results:**

Of 4437 identified studies, 12 were eligible. Intervention categories were case management (*n* = 2), financial incentivisation (*n* = 3), service change (*n* = 5), invitation (*n* = 1), and mixed (*n* = 1). Two RCTs showed a positive effect of a case-management approach; for one study there was 'high' risk of bias. For other interventions, diverse non-randomised studies were included with either positive or mixed findings and risk of bias ranging from 'moderate' to 'critical'.

**Conclusion:**

The study identified a scarcity of data and although a case-management approach shows promise there should be further robust and high-quality research.

## How this fits in

People living with severe mental illness experience inequalities in preventable physical ill health and premature mortality. The current evidence base on the effectiveness of interventions to increase access to, and uptake of, physical health checks has not been systematically reviewed. This systematic review identified 12 studies investigating interventions to increase access and uptake of physical health checks, reviews, and screening. Study types and interventions were heterogeneous, with concerns of risk of bias. However, two randomised controlled trials highlighted the benefits of a case-management approach to support access to physical health checks and cancer screening.

## Introduction

In the UK, people living with severe mental illness (SMI) includes those diagnosed with bipolar disorder, schizophrenia, and other psychoses.^1^ Globally, prevalence of both bipolar disorder and schizophrenia has increased over the past 30 years, with nearly 40 million people and approximately 24 million affected, respectively.^2^ People living with SMI can experience a reduced life expectancy, which may be 15–20 years lower than the general population.^3^ They face an increased risk of premature mortality (death before the age of 75 years) compared with the wider population.^4^ A major cause for this inequity is preventable physical ill health.^5^ General practice has an important role in supporting people living with SMI with their physical health.^6^

In the UK, people living with SMI are invited to take part in an annual physical health check, delivered in primary or secondary care.^7^ NHS England recommends a national annual uptake target of 75%.^8^ However, uptake for those on the SMI register in England was 55% in the third quarter of 2023/2024, and as low as 24% in some parts of the North West.^4^ In England, cancer screening for breast, colorectal, and cervical cancer is lower in patients with SMI than the general population.^9^ Internationally, there are other examples of physical health screening initiatives for people living with SMI, from novel tools for facilitating screening to complex health service interventions, and concerns about uptake of physical health checks, reviews, and screening is echoed in the literature.^10^

People living with SMI can experience service access barriers, including in appointment navigation.^11^ As the lived experience of psychosis involves coping with complexity and stress, navigating health systems can be challenging.^12^ Moreover, healthcare services can be fragmented.^11^ People may anticipate or experience ‘negative’ labelling in services,^13^ with stigma influencing help-seeking and service use.^13^ Loss of insight, a major symptom of psychosis, may also have an impact on help-seeking behaviour.^14^

Identifying effective interventions to increase access to and uptake of physical health reviews or screening in SMI is a timely research area. A Cochrane review from over a decade ago, explored interventions to encourage uptake of cancer screening in this population but did not identify any relevant studies.^15^ A more recent scoping review mapped 38 studies on interventions promoting utilisation of physical health checks, reviews, and screening for people with SMI, but did not systematically assess intervention effectiveness or quality, although a shortage of interventions and evaluation of their effectiveness was highlighted.^5^ Meanwhile, a realist review including 22 studies descriptively reported on intervention effectiveness but had a broad scope and outcomes, and the review is now outdated as searches were conducted in 2016.^10^ The current evidence base on the effectiveness of interventions that directly target access to, and uptake of, physical health checks has not been systematically reviewed.

The aim of the study was to systematically collate and assess the evidence to determine the effectiveness of interventions used to increase access to and uptake of physical health reviews and screening for people living with SMI.

## Method

The protocol is published on the International Prospective Register of Systematic Reviews (PROSPERO): CRD4202458492.^16^ This review is reported in line with the Preferred Reporting Items for Systematic Reviews and Meta-analysis (PRISMA).^17^

### Search strategy and selection criteria

Medline ALL (Ovid), Cumulative Index to Nursing and Allied Health Literature (CINAHL), and PsycINFO (Ovid) were searched during October and November 2024 for studies published from 2000 onwards. The search strategy (Supplementary Table S1) included free text and MeSH terms, informed by related published reviews.^5,10^ Reference lists of relevant reviews and included studies were also hand-screened.

Search results were stored and deduplicated in EndNote21.

### Study eligibility

Inclusion and exclusion criteria are summarised in Supplementary Table S2. Study designs eligible for inclusion were randomised controlled trials (RCTs), cluster RCTs, controlled pre-/post- (‘before and after’) studies, interrupted time-series studies, cohort and case–control studies, and cross-sectional studies. Studies needed a comparator or control group. Studies from high-income countries were included, as the authors’ understanding of the research issue is predominantly grounded in UK-based evidence and future practice recommendations will have a UK focus. Only studies published in English language were included.

The population of interest was adults aged ≥18 years with an SMI diagnosis. Although the definition of SMI varies internationally to focus the systematic review scope only studies using the UK definition of bipolar affective disorder, schizophrenia, and other psychoses^1^ were included, unless studies using other definitions reported results by individual diagnosis.

Interventions aiming to increase access to, or uptake of, physical health checks, reviews, and screening, including uptake of cancer screening, were included. These could be conducted in primary, secondary, or tertiary care settings. The main outcome was uptake, receipt, or completion of physical health checks or cancer screening. A broad range of intervention categories and outcomes were included as an initial scoping review had highlighted the limited variability of intervention types in this research area.

### Study screening and data extraction

Search results were screened in Rayyan.^18^ Both title and abstract and the full-text screening were conducted by one reviewer, with independent co-screening of 20% at each stage. Discrepancies were resolved through discussion, with involvement of a third reviewer if needed. Data were extracted by one reviewer using a pre-defined Excel database.

### Risk of bias

Risk of bias (RoB) was assessed using version 2 of the Cochrane risk-of-bias tool for randomised trials (RoB 2) for RCTs and an adaptation of Risk of Bias in Non-randomised Studies — of Exposures (ROBINS-E) and Risk of Bias in Non-randomised studies – of Interventions (ROBINS-I) tools for non-randomised studies.^19,20^ The ‘preliminary considerations’ section of ROBINS-E was applied to all non-randomised studies, assessing authors’ attempts to control for confounding. No further assessment was undertaken for studies deemed to be at ‘very high’ RoB in this preliminary assessment. For remaining non-randomised studies, the ROBINS-I tool was applied. This strategy was informed by another systematic review with heterogeneous data.^21^ All RoB assessments were independently conducted by two reviewers.

### Data synthesis

Meta-analysis was deemed inappropriate given substantial heterogeneity across studies, including in population types, interventions, study design, and outcomes. Instead, SWiM (Synthesis Without Meta-analysis) methodology was applied,^21,22^ with iterative study grouping by intervention type. A summary of intervention effects from each study by outcome was compiled, with study ranking according to RoB. The standardised metric was an odds ratio (OR) as this was commonly used or readily calculated from study data. An effect direction plot was used to depict an overall positive (beneficial) or negative (harmful) effect per study.^23^ Where possible, adjusted estimates were prioritised over unadjusted estimates in data synthesis.

## Results

### Description of studies

**Figure 1.**
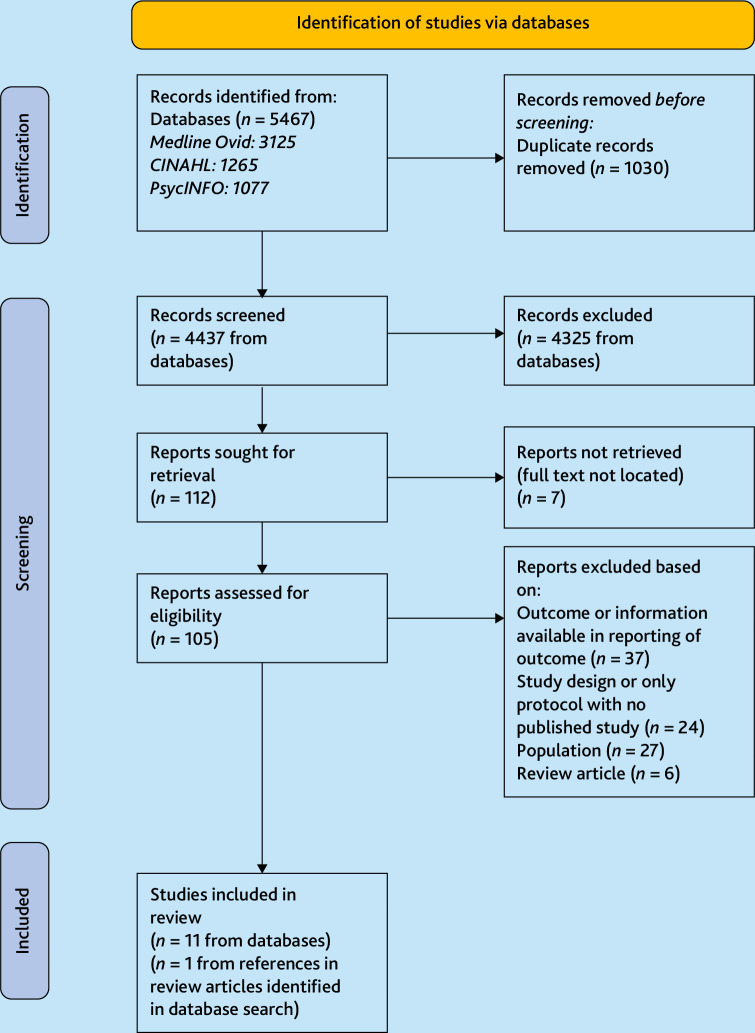
PRISMA flow chart.^17^

Following deduplication, 4437 results were available from database searches. After title and abstract screening, 112 reports were sought for full-text screening. In total 12 studies were included (Figure 1).

Study characteristics are included in Supplementary Table S3. This review includes two RCTs,^24,25^ two cohort studies,^26,27^ two combined cross-sectional and cohort studies,^28,29^ one combined cohort and interrupted time-series analysis,^30^ one interrupted time-series analysis,^31^ one pre-/post-feasibility study,^32^ and three cross-sectional study designs.^33–35^ Sample sizes ranged from 172 to 2 079 306. Most studies were conducted in the UK (*n* = 8), followed by the US (*n* = 3), and Japan (*n* = 1). Most were published within the past decade (*n* = 9). Six studies were set in primary care, four in psychiatric or mental health services, one in a behavioural health clinic, and one in veteran healthcare services.

### Intervention types

Interventions were categorised into broad types, including service change (*n* = 5),^26,31–34^ financial incentivisation (*n* = 3),^28–30^ case management (*n* = 2), invitation (*n* = 1),^35^ or mixed (*n* = 1).^27^ Supplementary Table S4 summarises the intervention elements in each study. Most commonly, interventions were targeted at healthcare professionals (*n* = 9),^24–30,32,34^ or at the organisational level (*n* = 7),^27–33^ with some interventions, such as financial incentivisation, spanning both these levels. Four interventions were targeted at service users.^24,27,31,35^

Several studies included education, support, or behavioural change targeted at the service user,^24,27,31^ or healthcare professional,^25–27,32,34^ with other elements including service navigation^24,27^ or peer support.^27^ Other studies included service/process adaptation such as invitations,^25,31,34,35^ integration of physical health assessments into another service,^26,27,31,32^ colocation of physical health services with other services,^27,31,33^ financial incentivisation,^28–30^ and coordination/communication between the base service and physical health services.^31,32^

### Outcome types

Supplementary Table S5 summarises study outcomes. Three studies reported receipt of/or participation in cardiovascular or cardiometabolic risk factor screening.^25,27,35^ Three studies reported receipt of a physical health check or assessment^26,32,33^ and another considered receipt of a ‘data-rich’ physical health check (versus lower-quality checks).^34^ Two studies measured change in utilisation of primary care and medical outpatient services or GP consultation rates.^30,31^ Two studies reported recording of alcohol consumption^28,29^ and one reported receipt of cancer screening.^24^

### Risk of bias

**Figure 2.**
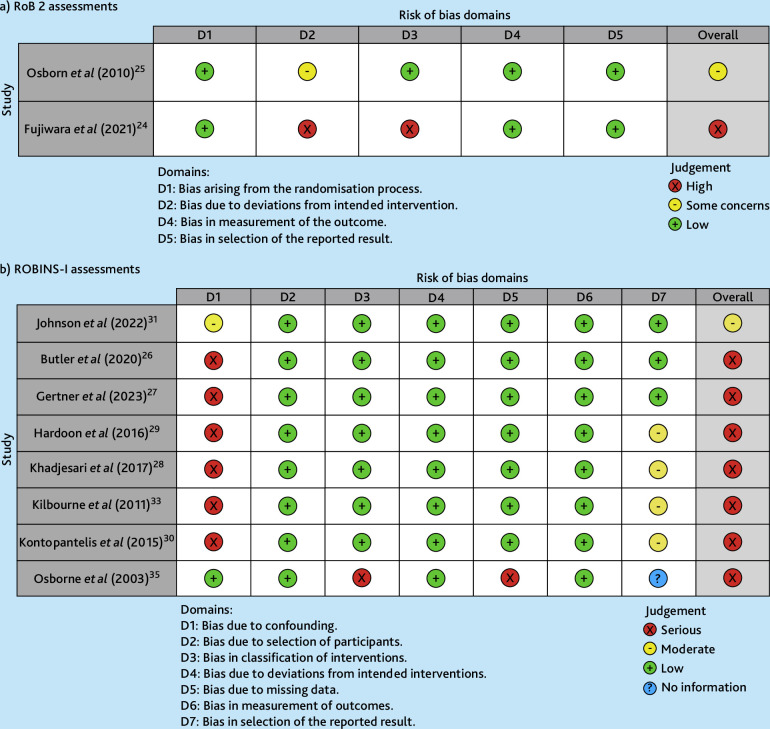
Traffic light plot of risk of bias (RoB) assessments. Assessments using a) version 2 of the Cochrane risk-of-bias tool for randomised trials (RoB 2) and b) Risk of Bias in Non-randomised Studies — of Interventions (ROBINS-I) .

Figure 2 summarises RoB assessments, with additional detail in Supplementary Tables S6 and S7. One RCT had ‘some concerns’ relating to information shortages.^25^ Another RCT had ‘high’ RoB because of concerns about group differences and missing data.^24^ One study that was an interrupted time series had ‘moderate’ RoB because of potential confounders.^31^ Seven studies of predominantly cross-sectional or cohort design had ‘serious’ RoB because of limited inclusion of potential confounders.^26–30,33,35^ Two studies (one pre-/post-feasibility study and one cross-sectional study) had ‘critical’ RoB, relating to risk of confounding.^32,34^

### Intervention effectiveness

This is reported in the summary of outcome data (Supplementary Table S5) and effect direction plot (Supplementary Figure S1). The findings are presented according to intervention type.

#### Case management

Two RCTs, one on healthcare professional support for patients with service/appointment navigation^24^ and one on physical health screening promotion,^25^ reported positive effects in the receipt of cancer screening and physical health checks, respectively (OR 6.67, 95% confidence interval [CI] = 3.04 to 14.62) and OR 5.2 (95% CI = 1.8 to 15.3). Of note, Fujiwara *et al* had a ‘high’ RoB,^24^ and Osborn *et al*, although only having ‘some concerns’ of bias, had slight differences in gender split between groups (intervention group 34% women versus 45% in the comparator group).^25^

An additional study^27^ used care coordination as part of a wider ‘enhanced primary care’ approach to increase cardiometabolic screening. Compared with usual care, there was increased receipt of three of four constituents of cardiometabolic screening.

#### Financial incentivisation

Three UK-based studies investigated the impact of the Quality and Outcomes Framework (QOF) payment framework.^28–30^ In two studies with ‘serious’ RoB, alcohol consumption recording for patients with bipolar disorder was increased versus a control group (adjusted risk ratio [RR] 4.45, 95% CI = 4.15 to 4.77, *P*<0.001)^29^ and for patients with schizophrenia (adjusted RR 3.8, 95% CI = 3.54 to 4.08).^28^ In another study with ‘serious’ RoB,^30^ post-QOF GP face-to-face consultation rates appeared to increase in the SMI group versus control group, although not after adjusting for confounders.

#### Service change

There were five service-change studies;^26,31–34^ these were diverse in study design and intervention. Four included interventions that tried to narrow the gap between physical health care and other services. In a study integrating a behavioural health clinic with primary care, the intervention produced positive effects in primary care service utilisation, with ‘moderate’ RoB concerns.^31^ In a study with ‘serious’ RoB and set in veteran healthcare services with approximately 90% men in both groups,^33^ colocation of physical and mental health services improved receipt of some but not all physical health checks and cancer screening. In a study with ‘critical’ RoB, involving an integrated care pathway combining physical and mental health support,^32^ there was no change in receipt of an eight-point physical health check. In a study with ‘serious’ RoB that involved providing point-of-care blood tests in an early intervention service for psychosis,^26^ receipt of physical health checks appeared to increase in the intervention group; however, this was non-significant when adjusting for increasing historical completion rates.

Another service-change intervention with ‘critical’ RoB produced positive effects.^34^ This involved implementation of a computerised cardiovascular screening template, increasing receipt of ‘data-rich’ physical health checks versus lower-quality physical health checks when comparing intervention and control sites (OR 2.92, 95% CI = 2.13 to 4.01).

#### Invitation

One study with ‘serious’ RoB^35^ used a letter and telephone invitation approach to try to increase cardiovascular risk screening for people with psychosis. An adjusted OR indicated no difference in screening uptake between groups, although authors reported increased GP consultation rates in the intervention group compared with the control population.

#### Mixed

In a study with ‘serious’ RoB, an ‘enhanced primary care’ approach involved care coordination, peer support, self-management, and links with behavioural health teams.^27^ Compared with usual care, enhanced primary care increased receipt of three of four constituents of cardiometabolic screening, including HbA1c screening that was increased by 18 percentage points (95% CI = 10 to 25). Of note, the intervention group had a higher proportion of men than the comparator group (60% versus 35%).

## Discussion

### Summary

Twelve studies of diverse intervention types with varied outcomes were identified. Two RCTs demonstrated the benefits of a case-management approach in supporting patients with physical health or cancer screening.

QOF payments produced positive effects in increasing recording of alcohol information, highlighting this as an intervention warranting further investigation. However, there was a mixed impact on GP consultation rates. Service-change interventions were diverse in design, with positive or mixed effects.

A multicomponent ‘enhanced primary care’ intervention increased receipt of most but not all elements of cardiometabolic screening. An invitation approach had a limited impact on cardiovascular risk screening.

### Strengths and limitations

A comprehensive search was conducted, although this could have been strengthened by reviewing grey literature, searching more than three databases, and inclusion of non-English language papers. However, given the mixed evidence base it is unlikely that including further unpublished studies would have altered the conclusions. Intervention components were categorised, providing a comprehensive overview to date.

Although both title and abstract and full-text screening were conducted, this was predominantly by one reviewer, with 20% of screening being conducted by a second reviewer. The RoB of included studies was also rigorously assessed, and, although a meta-analysis was deemed inappropriate, the use of ‘SWiM’ methodology enabled systematic, transparent, and reproducible data synthesis.

This review was restricted to studies from high-income countries to ensure the findings are applicable to the UK context. However, the authors of the current study recognise that this may limit generalisability of the findings to other, particularly middle-/low-income, settings. Given that limited interventions were identified in an initial scoping review, studies from primary, secondary, and tertiary care were included. However, this makes the extrapolation of the current study’s findings to general practice challenging.

The available studies were heterogeneous in study design, interventions, outcomes, and findings. Only two studies were RCTs, with one study having ‘high’ RoB. Of note, a cluster RCT was included, although this was for a feasibility study.^25^ The trial met the study’s inclusion criteria and, although the sample size was relatively small (*n* = 327), the RoB was defined as ‘some concerns’, while other studies included in the review had higher RoB. The design of some of the included studies was weak for assessing intervention effectiveness (for example, cross-sectional studies with a comparator group) but were included to provide a comprehensive summary of the limited evidence base. Of the 10 non-randomised studies, eight adjusted for confounding.

Another limitation is that some studies used a ‘proxy’ to investigate engagement with physical health care, for example, rate of GP consultations, potentially risking measurement error. Another study^34^ assessed completion of ‘data-rich’ physical health checks with the alternative outcome still including a degree of physical health check. This contrasted with other studies where the outcome of a physical health check was binary (present or absent), challenging data synthesis. Finally, in the studies measuring the impact of QOF, the intervention changed over time, challenging direct pre-/post-intervention comparisons.

### Comparison with existing literature

The current study's finding of varying evidence is consistent with prior literature reviews,^5,10^ including the recent scoping review of 38 studies and the realist review of 22 studies, with both reporting a shortage of robust evaluations and research quality challenging reporting of an effect size.^5,10^ Moreover, a Cochrane review exploring interventions to increase uptake of cancer screening for people living with SMI did not identify any relevant RCTs.^15^

Case management has previously been identified as a strategy to enhance contact with psychiatric services for people with SMI.^36^ Support with service navigation and care coordination was central to two studies using a case-management approach in this review.^24,27^ Care navigation has been identified as important in improving diabetes management for people with both SMI and diabetes, including in attending essential health checks.^37^

### Implications for research and practice

Improving physical health checks for people with SMI in primary care is endorsed in high-level UK policy, such as Core20plus5.^1^ In this framework for action, NHS England provides recommendations for physical health checks in primary care, and guidance for personalised follow-up support as part of ‘don’t just screen, intervene’. The NHS Long Term Plan also includes a commitment to improvement in this area and National Institute for Health and Care Excellence guidance stipulates the importance of having SMI registers in general practice.^1,38^ When addressing health inequalities more generally, GP teams can also consider a broader action framework across multiple domains including organisational practices, for example, flexibility in access to care for service users.^39^

GP teams can support patients living with SMI and their families over time.^6^ The finding of positive effects with a case-management approach, where service users are individually supported with physical health checks, is in keeping with this premise. However, two studies included in this review using this approach were set in non-general practice settings, challenging applicability and highlighting a need to assess these interventions in primary care. Likewise, service-change interventions included in this review were predominantly set in non-general practice settings (for example, in mental health or behavioural health services), limiting interpretation of these results in general practice. This could be an area for further exploration, including how general practice can collaborate with other health service areas to improve physical health care and chronic disease management for people living with SMI.

Given the scale of the problem, investment in physical health care for people living with SMI is justifiable in general practice.^40^ Indeed, this systematic review highlights financial incentivisation in general practice, particularly in the form of QOF payments, as producing positive effects in the recording of service users’ health information. However, in the context of resource constraints, the authors of the current study note recent reductions in QOF payments as part of the changes to the GP Contract in 2025/2026 affecting all GP practices in England.^41^

Overall, this systematic review highlights a need to conduct more high-quality research on this topic, with involvement of service users. Building the evidence base in general practice is essential, since current studies include several non-general practice contexts. Although more RCTs assessing intervention effectiveness are needed, for some intervention types an RCT design may not be feasible or appropriate, and intervention complexity may challenge formal evaluation. For example, service-change or mixed-component initiatives may represent complex interventions requiring comprehensive evaluation processes and considering broader influences such as structural or political factors. Qualitative research considering patient and healthcare professional perspectives on barriers and enablers to improving physical health for people living with SMI is also important. As well as improving participation in health checks for people living with SMI, additional research could consider whether the strategy of physical health checks meaningfully improves outcomes and reduces disparities.

The conclusions are limited by a paucity of evidence, as well as concerns of potential biases in the available evidence. The findings suggest that a case-management approach could be a promising intervention; however, the authors support calls for a more robust evidence base in this area, to address the concerning health inequalities experienced by people living with SMI.

## Data

Available in the main findings section, as well as in the supplementary material file.
